# Noble-Metal Nanoparticle-Embedded Silicon Nanogratings via Single-Step Laser-Induced Periodic Surface Structuring

**DOI:** 10.3390/nano13081300

**Published:** 2023-04-07

**Authors:** Yulia Borodaenko, Evgeniia Khairullina, Aleksandra Levshakova, Alexander Shmalko, Ilya Tumkin, Stanislav Gurbatov, Aleksandr Mironenko, Eugeny Mitsai, Evgeny Modin, Evgeny L. Gurevich, Aleksandr A. Kuchmizhak

**Affiliations:** 1 Institute of Automation and Control Processes, Far Eastern Branch, Russian Academy of Sciences, 690041 Vladivostok, Russia; 2 Institute of Chemistry, Saint Petersburg State University, 7/9 Universitetskaya Nab., 199034 St. Petersburg, Russia; 3 Interdisciplinary Resource Center for Nanotechnology of Research Park of SPbSU, Saint Petersburg State University, 7/9 Universitetskaya Nab., 199034 St. Petersburg, Russia; 4 Institute of Chemistry FEB RAS, 690022 Vladivostok, Russia; 5 CIC nanoGUNE BRTA, E-20018 Donostia-San Sebastian, Spain; 6 Laser Center (LFM), University of Applied Sciences Munster, Stegerwaldstraße 39, 48565 Steinfurt, Germany; 7 Far Eastern Federal University, 690090 Vladivostok, Russia

**Keywords:** direct laser nanostructuring, femtosecond pulses, laser-induced periodic surface structures, laser-induced decomposition, nanoparticle formation, hybrid nanostructures

## Abstract

Here, we show that direct femtosecond laser nanostructuring of monocrystalline Si wafers in aqueous solutions containing noble-metal precursors (such as palladium dichloride, potassium hexachloroplatinate, and silver nitrate) allows for the creation of nanogratings decorated with mono- (Pd, Pt, and Ag) and bimetallic (Pd-Pt) nanoparticles (NPs). Multi-pulse femtosecond-laser exposure was found to drive periodically modulated ablation of the Si surface, while simultaneous thermal-induced reduction of the metal-containing acids and salts causes local surface morphology decoration with functional noble metal NPs. The orientation of the formed Si nanogratings with their nano-trenches decorated with noble-metal NPs can be controlled by the polarization direction of the incident laser beam, which was justified, for both linearly polarized Gaussian and radially (azimuthally) polarized vector beams. The produced hybrid NP-decorated Si nanogratings with a radially varying nano-trench orientation demonstrated anisotropic antireflection performance, as well as photocatalytic activity, probed by SERS tracing of the paraaminothiophenol-to-dimercaptoazobenzene transformation. The developed single-step maskless procedure of liquid-phase Si surface nanostructuring that proceeds simultaneously with the localized reduction of noble-metal precursors allows for the formation of hybrid Si nanogratings with controllable amounts of mono- and bimetallic NPs, paving the way toward applications in heterogeneous catalysis, optical detection, light harvesting, and sensing.

## 1. Introduction

Catalytic applications of nanoparticles (NPs) made of typical noble metals (such as silver, palladium, and platinum) or their alloys have always attracted considerable research attention [1,2,3,4]. Due to the high surface-to-volume ratio and high surface energy, atoms on metal NPs’ surfaces are catalytically active for a number of heterogeneous processes, such as hydrogenation, C-C coupling, carbonylation, oxidation, etc. [5,6,7,8] However, high NP activity also causes their aggregation and deactivation by secondary nucleation and recrystallization, which is a serious disadvantage when used in practical catalytic reactions and, therefore, limits industrial application [9,10]. One of the widely used strategies to stabilize metal NPs is their encapsulation on solid supports, which not only prevent NP migration and coalescence but also can significantly increase the catalyst recycling accessibility [1,10,11,12] and, in some cases, owing to the strong interactions between metal NPs and supports [13,14], can modify electronic properties of metal NPs and give rise to catalytic performance.

Numerous types of micro- and nanostructured materials with large surfaces hosting catalytically active NPs can play the role of a solid support. Common examples include metal or non-metal oxides [1,2,15], semiconductors [8,16], carbon [17], or functionalized polymers [18,19,20]. Specifically, for catalytic reactions driven by sunlight radiation, the realization of light-absorbing supports is highly demanded. Such “ultra-black” surfaces provide a convenient pathway to trap and enhance broadband optical radiation within the functional surface layer loaded with noble-metal NPs, facilitating their catalytic performance [21,22,23,24,25]. The mechanism is based on light localization by nanostructures made of dielectric and semiconductor materials characterized by rather low optical losses and high refractive index such as Si, Ge, TiO_2_, etc.

Large specific surface areas and light-trapping performance are known to appear for surfaces containing carefully elaborated nanomorphologies, which highlights the practical importance of flexible and inexpensive nanostructuring technologies. Beyond common lithography-based technologies, which usually involve multi-step and time-consuming processes, direct surface nanostructuring with pulsed laser radiation appears to be a versatile and green technology allowing for the formation of various functional surface morphologies and nanostructures yet a lower spatial resolution. Ultrashort (femtosecond; fs) laser radiation is beneficial for precise material nanostructuring as temporally confined laser energy ensures delicate material ablation at minimized heat-affected zones. Single- and multi-pulse laser exposure can also initiate self-organization phenomena, resulting in the formation of chaotic or regular surface morphologies. Laser-induced periodic surface structures (LIPSS) represent a common example of a light-driven self-organization that, in most cases, originates from the interference phenomena between incident light and surface waves and results in the formation of grating-type surface morphologies oriented in either the perpendicular or polarization directions [26,27]. The utilization of structured laser beams for material texturing [28] permits the control of intensity, phase, and polarization distribution within the laser focal spot, allowing for the formation of more complicated irregular naturally inspired nanomorphologies that are of great interest for various applications including metasurfaces with anisotropic optical responses, promotion of cell adhesion, and wetting control [29,30,31,32].

At the same time, along with local ablative material removal from the exposed surface sites, intense laser radiation can be used as a versatile tool driving surface activation/functionalization for subsequent targeted deposition of the NPs via chemical binding [33] or various local photo- or thermally driven chemical reactions [16,21,34,35]. Such reactions can stimulate growth of the NP (including noble-metal ones) via decomposition of related salt/acid precursors within the laser focal volume [36]. Importantly, both the surface nanopatterning of the underlying substrate and its decoration with laser-synthesized NPs can be realized in one pot within an easy-to-implement fabrication cycle in sharp contract to laser-induced functionalization requiring additional steps such as surface texturing and NP synthesis that elongate the production chain, making the resulting structures rather expensive. To date, few papers reported that fs laser texturing of monocrystalline silicon wafers placed in a liquid solutions containing noble-metal ion precursors were found to yield a nanoscale surface morphology simultaneously with its decoration by metal NPs [16,37,38,39]. Such a single-step fabrication procedure is flexible regarding the ability to control both the Si surface nanomorphology (i.e., specific surface area and optical properties), as well as the degree of its loading with the noble-metal NPs, thus being practically attractive for various applications including optical sensing, light harvesting, photodetection, and heterogeneous catalysis. Meanwhile, the mentioned studies mainly aimed to produce nanotextured semiconductor surfaces decorated with common noble-metal NPs (such as silver and gold), leaving behind other practically relevant mono- and multi-metallic combinations of the decorating NPs. Moreover, all the mentioned previous studies did not consider the application of structured light for surface nanotexturing together with laser-driven chemistry.

In this paper, we report the single-step fabrication of Si nanogratings with a periodicity below 250 nm decorated with mono- (Pd, Pt, and Ag) and bimetallic (Pd-Pt) nanoparticles NPs. Such a direct maskless method involves laser-induced periodic surface structuring of commercial monocrystalline Si wafers in aqueous solutions containing related salt/acid precursors such as palladium dichloride, potassium hexachloroplatinate, and silver nitrate, or even their mixture. The thermal decomposition of the metal precursors localized at the laser-exposed Si interface creates the NP embedded in the nanograting trenches, while the laser processing parameters allow for the control of the general surface morphology, nanograting orientation, and amount of decorating noble-metal NP. The produced hybrid Pd-Si nanogratings with radially varying nano-trenches were found to demonstrate strong anisotropic light-trapping characteristics, as well as photocatalytic activity, confirmed by SERS tracing of the dimerization of para-aminothiophenol (PATP) to dimercaptoazobenzene (DMAB).

## 2. Materials and Methods

### 2.1. Materials

Palladium dichloride PdCl_2_ (99.9%), potassium hexachloroplatinate K_2_[PtCl_6_] (99.9%), and silver nitrate AgNO_3_ (99%) were purchased from Sigma Aldrich and used as received without purification. Palladium chloride was dissolved in hydrochloric acid in a stoichiometric ratio (PdCl_2_ + 2HCl = H_2_PdCl_4_) to convert it into a water-soluble form. A 100 mM water-based solution of H_2_PdCl_4_ was used as the stock solution. Milli-Q water was used throughout all experiments.

### 2.2. Laser-Assisted Fabrication of Nanoparticle-Decorated Si Nanogratings

Direct laser nanopatterning of monocrystalline Si wafers was carried out with second-harmonic (515 nm) femtosecond (230 fs) laser pulses generated by a regeneratively amplified Yb:KGW-laser system at a 1 KHz repetition rate (Pharos, Light Conversion). A Si wafer was placed into a quartz cuvette that can be filled with various working solutions containing noble metal precursors such as H_2_PdCl_4_, AgNO_3_, or K_2_[PtCl_6_]. The laser radiation was focused through the few mm thick static working solutions onto the Si wafer surface using a dry microscope objective with a numerical aperture NA = 0.13. In all the experiments, laser fluence was kept below the single-pulse Si ablation threshold (F = 0.13 J/cm^2^) and controlled by a pyroelectric detector. Laser nanostructuring was performed either with a Gaussian-shape beam generated by the laser system or by radially (azimuthally) polarized vector beam with a donut-shape intensity profile. Such a beam was generated by passing the output laser beam through the commercial radial polarization converter (s-waveplate) [40].

### 2.3. Structural Characterization of Nanoparticle-Decorated Si Nanogratings

The morphology of the Si nanogratings was studied using scanning electron microscopy (SEM; Carl Zeiss Ultra 55+, Oberkochen, Germany) equipped with a calibrated energy-dispersive X-ray (EDX) detector for chemical composition characterization. EDX studies were carried out at 5 kV acceleration voltage to decrease the penetration depth of the electrons to the Si bulk and to enhance the signal from the near-surface layer containing noble-metal NPs. All samples were ultrasonicated in distilled water prior to analysis. An in-depth study of the Si nanograting morphology was realized by combining focused ion-beam (FIB; Helios 450, Thermo Fisher, Waltham, MA, USA) milling and transmission electron microscopy (TEM, Titan 300-60, Thermo Fisher). Cross-sectional cuts of the Si nanogratings were prepared with a Ga^+^-ion beam through the protective Pt overlayer.

### 2.4. Optical and Raman Characterization of Nanoparticle-Decorated Si Nanogratings

The anti-reflection performance of the metal-decorated Si nanogratings was attested using a home-built optical setup consisting of an optical microscope that was coupled with a grating-type optical spectrometer (Shamrock, Andor Technologies, Belfast, UK). Linearly polarized broadband radiation from the supercontinuum light source was used to probe the surface reflectivity. The optical signal was collected by a dry microscope objective with a numerical aperture of 0.95 and analyzed with a thermoelectrically cooled CCD camera (Newton, Andor Technologies). The reflectance spectra were averaged over different surface sites and different orientations of the laser-patterned area containing anisotropic Si nanogratings with respect to the polarization direction of the incident broadband radiation.

SERS spectra were acquired using a micro-Raman setup (Spectra II, NT-MDT) with an optical spectrometer equipped with a CCD-camera (i-Dus, Andor Technologies). The SERS signal was excited with a CW laser source at 473 nm pump wavelength. The laser radiation was focused onto the sample surface with a dry microscope objective (NA = 0.7; 100× Mitutoyo Plan Apo). The intensity of the circularly polarized laser radiation at the objective output was controlled by a power meter. Para-aminothiophenol (PATP) was used as a model molecular analyte for probing the photocatalytic activity of the NP-decorated Si nanogratings. The alcoholic solution containing 10^−6^ M of PATP was drop-casted over the Si wafer containing the laser-textured areas, while the SERS experiments were carried out after the drop evaporation and washing the sample with distilled water. Finite-difference time-domain (FDTD) simulations were carried out to reveal the local structure of the electromagnetic fields near the NP-decorated Si nanogratings upon their excitation at 473, 532, and 633 nm with linearly polarized plane waves. The simulation of the normalized electric-field amplitude (E/E_0_; E_0_ is an amplitude of the incident field) was undertaken with a commercial software package (Lumerical Solutions, Ansys, Canonsburg, PA, USA) considering the computation volume limited by periodic boundary conditions and perfectly matched layers in the lateral and vertical directions, respectively.

## 3. Results

**Figure 1:**
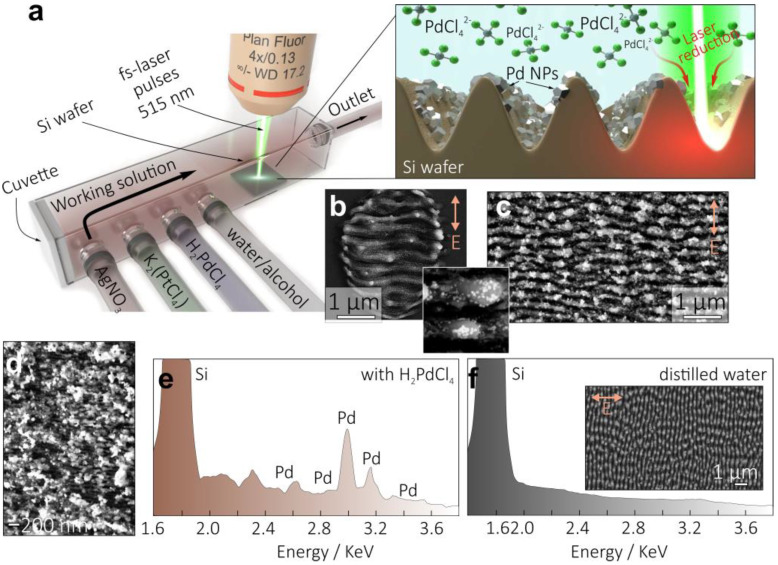
(**a**) Schematic representation of the Si wafer texturing in the quartz cuvette filled with various working solutions, as well as (right panel) a sketch illustrating formation of Si nanograting decorated with Pd NPs. (**b**) Top-view SEM image of the local surface modification produced on the Si surface upon its irradiation with 10 consecutive laser pulses at *F* = 0.1 J/cm^2^. Bottom inset provides a close-up image of the decorating Pd NPs. The aqueous solution contained 10^−3^ M of H_2_PdCl_4_. (**c**) Similar SEM image showing Si nanograting decorated with Pd NPs produced at *F* = 0.1 J/cm^2^ and scanning the sample surface at *v* = 100 µm/s and pulse repetition rate *k* = 1 KHz. (**d**) SEM image of the Pd-decorated Si nanograting with a period of 70 nm produced at N = 1000. (**e**,**f**) EDX spectra of the Si nanogratings produced in aqueous solution containing 10^−3^ M of H_2_PdCl_4_ (**e**) and in pure distilled water (**f**). Inset in (**f**) shows the SEM image of the Si nanograting morphology without noble-metal NPs. Arrows in all SEM images indicate polarization direction.

Figure 1a schematically demonstrates the experimental procedure of fs-laser nanostructuring of the Si wafer in the working solutions containing metal precursors. Let us first discuss the features of the laser material processing in the liquid environment. Taking into account transparency of the working solutions at 515 nm, absorption of the laser radiation by Si material causes strong heating. In turn, this initiates heating and boiling of the working solution at the interface. Excessive boiling leads to deterioration of the laser intensity distribution, resulting in stochastic behavior of the surface nanostructuring. This feature requires careful optimization of key laser processing parameters: laser fluence *F*, as well as number of laser pulses *N* applied per surface site.

Systematic studies showed that, under multi-pulse exposure, the use of *F* > 0.15 J/cm^2^ results in excessive boiling of the working solution, while laser fluence below 0.075 J/cm^2^ causes no morphology modification of Si wafer even under large number of laser pulses applied per spot (*N* > 1000). This generally defines a rather narrow processing window of Si wafers in working solutions allowing for the optimization of other relevant experimental parameters. The single-pulse fs-laser ablation threshold of monocrystalline Si in distilled water was previously measured to be about 0.13 J/cm^2^ at 515 nm laser wavelength [41]. Such fluence under single-pulse exposure causes only a shallow amorphisation of the near-surface Si layer. Meanwhile, consecutive irradiation of the surface by multiple laser pulses promotes morphological evolution via local melting as the amorphous Si absorbs the incident laser radiation more efficiently compared with the crystalline one [42]. Moreover, photoexcitation of the Si surface provides the conditions for coupling of the incident radiation to transient surface plasmon waves [43,44]. Interference of the incident and surface waves at the timescale that corresponds to the pulse duration creates periodic modulation of the intensity distribution at the solution–Si interface [22,45,46]. Laser fluence in the interference maxima can exceed the ablation threshold, resulting in imprinting of a grating-type surface morphology (also referred to as LIPSSs) or can generate these structures by inducing hydrodynamic flow driven by the periodically modulated surface temperature [47].

The result of the Si surface irradiation in aqueous solution containing 10^−3^ M H_2_PdCl_4_ with 10 consecutive fs-laser pulses (*N* = 10) at below-threshold fluence of *F* = 0.1 J/cm^2^ is shown in Figure 1b, revealing the formation of the nanograting with a characteristic period of 250 nm and a trench orientation that is perpendicular to the polarization of the incident radiation. By scanning the Si wafer surface with a laser beam and keeping the laser fluence *F* = 0.1 J/cm^2^ and *N* within the range of 8 to 30, one can cover larger surface areas with the nanogratings (Figure 1c). Noteworthily, a larger number of applied laser pulses per site deteriorates the regularity of the formed nanograting, while excessively large *N* > 1000 allows for the Si nanograting period to be observed down to 70 nm (Figure 1d). This feature was recently explained by the hybrid mechanism combining the formation of the initial nanograting with a period of 250 nm via the described interference effects and subsequent period downscaling via development of the hydrodynamic Rayleigh–Taylor instability in the melted Si layer [41].

**Figure 2:**
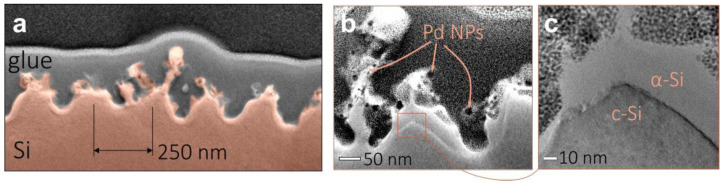
(**a**) SEM image of the cross-sectional cut of the Si nanograting morphology (*F* = 0.1 J/cm^2^, *v* = 100 µm/s, *k* = 1 KHz) decorated with Pd NPs. (**b**) Close-up TEM image of the isolated surface protrusions with the Pd NPs. (**c**) TEM image showing amorphisation of the textured Si surface.

A closer look at the nanograting morphology (see inset in Figure 1b) also reveals the formation of the Pd NPs decorating the Si nanograting. Such NPs exhibit the size ranging from 5 to 25 nm and are almost absent outside the laser exposed area, indicating the local nature of the laser-induced reduction process. EDX studies confirmed the formation of Pd NPs on the Si nanogratings (Figure 1e). Noteworthily, NP formation weakly deteriorates the Si nanograting formation as it was confirmed by similar experiments performed with pure distilled water used as a working solution. Laser texturing in this case produced a similar nanograting without any decorating NPs (Figure 1f). FIB milling was carried out to fabricate cross-sectional cuts of the Si nanograting morphology further visualized with TEM (Figure 2a–c). TEM images revealed the developed surface morphology of the nanogratings with the averaged trench height of around 250 nm, as well as multiple Pd NPs decorating the nanograting interface. Some of the NPs are seen to be partially embedded into the Si trenches, ensuring the good mechanical stability of such a hybrid Pd-Si morphology. Partial amorphisation of the Si material resulting from laser exposure can be also seen from the close-up TEM images (Figure 2c).

Uniquely, along with surface ablation (modification), laser radiation can deliver photons as well as heat to the working solution, stimulating both thermal and photochemical processes [48,49,50]. The key feature distinguishing this procedure from common chemical synthesis is localization of the reactions within a micron-scale focal spot area. An optical breakdown is a well-established mechanism of metal ion reduction under the action of intense laser pulses [51,52]. This process is accompanied by the formation of highly active unstable species such as radicals and solvated electrons. The former acts as a reducing agent for metal ions, and then reduced neural atoms aggregate, forming the NPs. This common mechanism works for a wide range of metal ions (Ag^+^, Cu^2+^, etc.); however, in the case of [PdCl_4_]^2−^, especially at relatively low laser intensity, different processes may be taken into account. The solution of H_2_PdCl_4_ exhibits a weak absorption band around 400 nm [53,54]. This band corresponds to d-d transition, which is known to be photo inactive and leads to internal conversion of the excited state back to the ground state [55]. In turn, under UV exposure, Pd^2+^ tends to be reduced by subtracting H from alcohols [56,57]; this process can be described by the following equation [58,59].

[PdCl4]^2−^ + RCH_2_OH + *hv* → Pd^0^ + R′CHOH + H^+^ + 4Cl^−^

At the same time, working solutions under study containing metal precursors are completely transparent at 515 nm, thus making photo-induced channels of the molecular precursor reduction inefficient. In this respect, considering that silicon melting occurs at 1410 °C, the thermal reduction of palladium chloride proceeding in a temperature range of 600–800 °C is a more convincing scenario of Pd NP formation [60,61,62,63,64]. One possible reaction that describes this process is as follows:

H_2_PdC_4_ = Pd + 2HCl + Cl_2_

**Figure 3:**
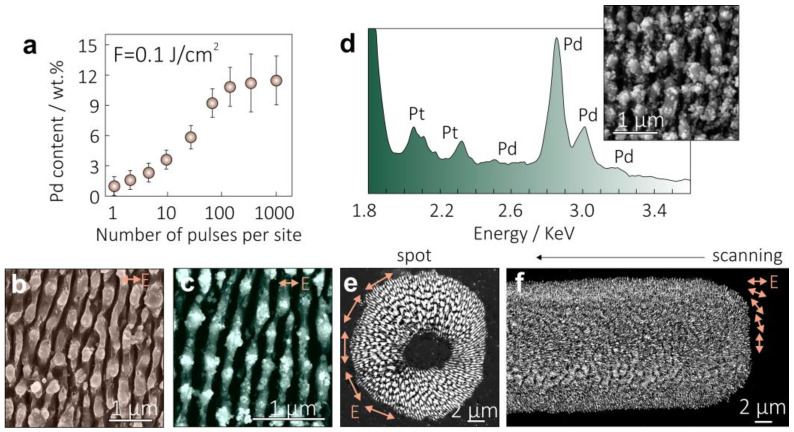
(**a**) Averaged amount of Pd in the Si nanogratings as a function of the number of laser pulses *N* applied per surface site. Pd content was averaged over 10 different surface sites produced at identical conditions. Experiments were carried out at fixed laser fluence *F* = 0.1 J/cm^2^ and working solutions containing 10^−3^ M of H_2_PdCl_4_. (**b**,**c**) Representative SEM images of Si nanogratings decorated with Pt and Ag NPs, respectively. (**d**) EDX spectrum of the Si nanogratings produced in aqueous solution containing 10^−3^ M of H_2_PdCl_4_ and 10^−4^ M of K_2_[PtCl_6_]. (**e**,**f**) SEM images of Pd-decorated Si nanogratings with characteristic periods of 250 (**e**) and 70 nm (**f**) recorded with an azimuthally polarized donut-shaped laser beam at *F* = 0.1 J/cm^2^. The 250 nm period nanograting was produced by applying *N* = 25 laser pulses per surface site, while the 70 nm period was produced by scanning the sample surface at *v* = 1 µm/s at pulse repetition rate of 1 KHz. Arrows in all SEM images demonstrate the polarization direction.

The laser-generated heat is efficiently localized at the solution–material interface, ensuring targeted formation of the Pd NPs in the formed Si nanogratings. The amount of metallic palladium depends on the number of laser pulses applied per surface site *N*, which was systematically studied with an EDX method to assess the averaged Pd content on the Si surface processed using an aqueous working solution containing 10^−3^ M of H_2_PdCl_4_ and a laser fluence of 0.1 J/cm^2^ (Figure 3a). These data also show that the Pd NP formation saturates at *N* > 100, which can be related to the removal of the nanoparticles by subsequently incident laser pulses. A larger deviation of the average Pd content with increasing *N* also indicates increasing contribution of the uncontrolled NP growth processes (for example, random NP redeposition from the other surface sites or formation of the NP formed directly in the working solution) that is undesirable for practical applications. For a fixed number of applied pulses *N*, the amount of NP can be also controlled by the concentration of metal-containing precursors. However, an excessive amount of H_2_PdCl_4_ (more than 5 × 10^−3^ M) was found to make the reduction process less controllable, apparently owing to the formation of free Pd NPs in the working solution and their random redeposition onto the Si surface. The methanol working solution containing H_2_PdCl_4_ was also found to yield Si nanogratings decorated with Pd NPs. At the same time, organic solutions (such as methanol) cause a certain increase in the average carbon content from 3 to 5% in the formed Si nanogratings owing to laser-induced decomposition of the corresponding solvent molecules, as confirmed by the EDX studies.

Aqueous solutions containing other noble-metal precursors (AgNO_3_ and K_2_[PtCl_6_]) were also prepared and attested regarding the possibility of fabrication of other hybrid NP-decorated Si nanogratings. Both utilized salts are characterized by a rather low decomposition temperature below 250 °C, facilitating thermal reduction scenarios. SEM images on Figure 3b,c demonstrate the morphology of the Si nanogratings decorated with Ag and Pt NPs. For both noble-metal precursors, the formed NP demonstrates a broad size distribution ranging from 10 to 40 nm. Importantly, more complicated working solutions containing different metal precursors can be utilized to produce the nanogratings decorated with bimetallic NPs. Figure 3d demonstrates this remarkable modality, providing a reference SEM image of the Si nanograting simultaneously decorated with Pd and Pt NPs as well as corresponding EDX spectra of this hybrid multi-element structure. The structure was produced using typical laser processing parameters (*F* = 0.1 J/cm^2^, *N* = 25), as well as aqueous working solution containing 10^−3^ M of H_2_PdCl_4_ and 10^−4^ M of K_2_[PtCl_6_], resulting in averaged Pd and Pt contents of 7 and 3 wt.%, respectively, according to the EDX analysis performed. An adjustment of the metal precursor content in the working solutions allows for control of the resulting composition of such a hybrid multi-element surface morphology.

Since the plasmon-mediated interference phenomenon underlies the formation of Si nanogratings, their orientation is usually perpendicular with respect to the polarization vector of the incident laser radiation. Such an orientation also defines the orientation of short-period (70 nm) Si nanogratings formed under *N* > 1000, despite hydrodynamic instability being responsible for this surface morphology. Structured laser beams provide pathways to control both the intensity and polarization distribution, enabling the local orientation of the nanogratings to be defined in turn. To demonstrate the applicability of such an approach to creating NP-decorated Si nanogratings with a radial orientation of the nano-trenches, we used a cylindrical vector laser beam with a donut-shape intensity profile, as well as an azimuthal polarization distribution (see the Materials and Methods section). An example of the surface morphology formed in an aqueous working solution using such a structured beam (*F* = 0.1 J/cm^2^, *N* = 25) is provided in Figure 3e, revealing a ring-shape nanotextured area containing radially arranged 250 nm period Si nanogratings. Scanning the Si surface with such a beam along a linear trajectory allows for a nanotextured surface track to be produced with 70 nm period nanogratings with an orientation gradually rotating by 90° when moving from the center of the track towards its periphery (Figure 3f). Such modality allows for the creation of grating-type structures with isotropic optical properties since ordinary nanogratings with linear arrangement of the trenches expectedly exhibit anisotropic optical behavior [65,66,67,68]. The experiments with structured laser beams also clearly demonstrate that the beam shaping modality allows for the diversity of surface morphologies that can be produced via straightforward fs-laser patterning to be expanded.

**Figure 4:**
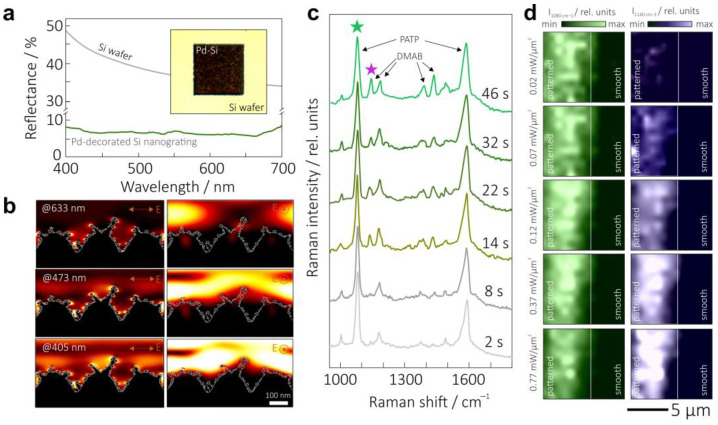
(**a**) Reflectance spectra of Pd-decorated Si nanograting and pristine Si wafer. The following parameters were used for grating fabrication: *F* = 0.1 J/cm^2^, *N* = 25, aqueous solution with 10^−3^ M of H_2_PdCl_4_. Inset shows the microscopic optical image of the laser-patterned (100 × 100 µm^2^) Si surface. (**b**) Normalized EM-field amplitude E/E_0_ in the vicinity of Pd NP-decorated Si nanograting excited by a plane wave with wavelengths of 405, 473, and 633 nm polarized perpendicular (left column) or parallel (right column) to the nano-trench orientation. (**c**) Representative series of time-resolved SERS spectra showing photocatalytic transformation of PATP to DMAB product upon continuous irradiation of the probe molecules adsorbed on the Pd-decorated Si nanogratings. Total laser exposure time is indicated near each spectrum. (**d**) Energy-resolved SERS maps (10 × 10 µm^2^) showing intensity distribution of PATP- (1080 cm^−1^) and DMAB-related (1140 cm^−1^) bands near the interface between the Pd-decorated Si nanogratings and smooth Si surface. The maps were measured at various laser intensities ranging from 0.02 to 0.77 mW/µm^2^ at an accuracy of 250 nm and signal accumulation time of 0.5 s per spot.

The fabricated anisotropic Si nanogratings decorated with noble-metal nanoparticles demonstrate remarkable antireflection performance, as revealed by the corresponding spectra shown in Figure 4a. In particular, the 250 nm period Si nanograting with decorating Pd NPs (around 6 wt.%) exhibited an average reflectivity of about 7% in the visible spectra range (400–700 nm), decreasing the reflectivity of pristine Si by almost an entire order of magnitude. A larger content of metallic NPs (up to 12 wt.%) was found to increase the average reflectivity of the decorated Si nanogratings by up to about 9–10%, which is related to the generally large reflectivity of the formed metal surface layer. Noteworthily, the average reflectivity does not change upon varying the polarization direction of the probing white light that confirms anisotropic optical response of the nanogratings. The laser-textured Si surface area appears black upon observation with an optical microscope, confirming a broadband antireflection behavior (inset of Figure 4a). A strong contribution of plasmon-mediated absorption from the Pd (as well as Pt) NP is expected in the UV spectral range; thus, the observed low visibility-range reflectivity can be generally related to the Si nanotexturing that reduces the refractive index jump at the air–material interface. Light scattering by the nanoscale surface features that is redirected along the surface can also contribute to the observed reflectivity drop. Such a nanograting morphology allows for the incident optical radiation in the gap between the NP-decorated nano-trenches to be trapped, facilitating huge enhancements in the local EM-field therein that is beneficial for optical sensing and heterogeneous catalysis driven by optical radiation. Light localization is more efficient when the polarization vector of the incident radiation is perpendicular with respect to the nano-trench orientation as it is illustrated by corresponding distributions of the normalized EM-field amplitude E/E_0_ calculated for a 250 nm period Si nanograting decorated with Pd NPs upon excitation at various visible-range pump wavelengths (Figure 4b).

To confirm the photocatalytic behavior of the hybrid Pd-Si nanotextured surface, we used, as a common indicator, the catalytic dimerization of the PATP molecules to DMAB products under optical radiation exposure [8,69]. The efficiency of this process can be monitored in situ by measuring the evolution of the SERS spectra of the pristine molecules and the product pumped by the same laser radiation (see the Materials and Methods section). In particular, pristine molecules are known to demonstrate two main Raman-active bands centered at 1078 and 1587 cm^−1^ [69], while four additional bands appear in the SERS spectra upon catalytic transformation of the analyte to the DMAB product. The series of SERS spectra in Figure 4c demonstrates such a transformation upon continuous laser exposure for 46 s. As can be seen, the intensity of the DMAB-related bands grows, indicating a gradually increasing amount of the product within the exposed area. Noteworthily, further laser irradiation leads to saturation of the photocatalytic transformation according to the dynamics of the related Raman bands since all the molecules within the probed laser spot were dimerized. Finally, even longer exposure times revealed continuous photodegradation of the product (not shown here). To study the PATP-to-DMAB photocatalytic conversion in more detail, SERS maps showing the surface distribution of the intensity of PATP- (1080 cm^−1^) and DMAB-related (1140 cm^−1^) bands were recorded by varying the intensity of the pump radiation. SERS maps were recorded near the interface between the laser-textured and pristine Si surface areas particularly to show the absence of any SERS signal and catalytic activity on the smooth Si surface containing no noble-metal NPs (Figure 4d). As can be seen, at low pump intensities, PATP-to-DMAB transformation occurs only at certain surface sites, demonstrating the strongest photocatalytic activity, apparently related to the random arrangement and amount of the Pd NPs decorating Si nanogratings. Once the pump intensity increases, a more uniform distribution of the intensity of the DMAB-related band (>0.12 mW/µm^2^) is observed, indicating uniform catalytic conversion over the Pd-decorated surface area. The uniform distribution of the intensity of PATP-related Raman band (left column in Figure 4d) also shows the corresponding uniform distribution of the pristine molecules over the textured surface. Moreover, this feature indicates a rather uniform SERS signal enhancement over the Pd-Si nanograting that is beneficial for reliable measurements. The observed SERS effect is related to the contribution of well-known chemical and electromagnetic enhancement mechanisms. Based on the difference between the Raman signal measured on laser-textured and smooth Si surfaces, the SERS enhancement factor for Pd-decorated Si nanograting functionalized by 10^−6^ M of PATP can be estimated to be about 10^5^. Evidently, larger SERS enhancement factors can be expected with laser radiation in the UV spectral range where decorating Pd NPs demonstrate stronger plasmonic response.

## 4. Conclusions

Ultrafast laser radiation can deliver spatially confined energy to simultaneously drive material ablation and local chemical reactions. This feature was used in this work to create a hybrid metal–semiconductor nanomorphology, demonstrating anisotropic broadband antireflection performance (average visible-range reflectivity about 7%) and photocatalytic activity. The morphology represents nanogratings with a characteristic periodicity below 250 nm formed on the surface of monocrystalline Si and decorated with mono- (Pd, Pt, and Ag) and bimetallic (Pd-Pt) NPs. Key laser processing parameters (such as fluence, number of applied laser pulses per surface site, and polarization distribution) allow for the morphology and orientation of the nanogratings, as well as amount of decorative NPs, to be controlled, making the technology attractive for various applications related to catalysis, sensors, photodetectors, and solar-light harvesting. Moreover, the produced Si nanogratings can be used as a support substrate for subsequent deposition of chemically synthesized NPs via covalent binding [33] or sputtered percolated films, expanding the range of potential applications.

## Data Availability

The data presented in this study are available from the corresponding author upon request.
